# Observations on the Nature and Treatment of Cholera, and on the Pathology of the Mucous Membranes

**Published:** 1829-01-01

**Authors:** 


### Observations on the Nature and Treatment of Cholera; and on the Pathology of Mucous Membranes. By *Alex. Turnbull Christie*, M.D. Madras Medical Establishment; and lately in Medical Charge of the Civil Department in the Southern Mahratta Country. Octavo, pp. 137. Edinb. and London, 1828.

The mucous membranes seem to be the popular favourites of the day; and as, by our early notices of Broussais, and other Continental writers, we were considerably instrumental in engendering this taste among British practitioners, we have, in some degree, entailed upon ourselves the duty, which we will cheerfully discharge, of ministering to its demands. The work, of which we are about to present an analysis to our readers, contains an application of the pathological state of these membranes, to the solution of the phenomena of the cholera of India; and, though the author has been anticipated in this application by writers, British and foreign, yet are his views sufficiently distinct from those of his precursors in the field, to merit considerable attention from the profession.

The first part of the work is on the "Pathology of the Mucous Membranes," which are liable, he conceives, to two morbid conditions: "*inflammation*, evinced by the following signs; viz. increased heat, pain, redness and swelling; and *catarrh*, characterised by the secretion of the membranes being depraved and increased in quantity." As it is from this second state that the author considers the phenomena of the Indian epidemic to have resulted; and as only by advocating its existence, independently of inflammation, is he distinguished from Gravier, and other writers of the school of Broussais, he shall be, on this point, his own interpreter.

"It has been stated as a general law, by several eminent authors, that inflammation of mucous membranes is accompanied by increased mucous secretion; and pathologists almost invariably attribute catarrh to an inflammation of the mucous membrane in which it occurs.* This I apprehend, is far from being correct; for are there not numerous examples of inflammation of a mucous membrane without increased secretion, and of catarrh without inflammation? We have examples of the former in ophthalmia, inflammatory sore throat, some cases of gastritis, and perhaps also of enteritis: of the latter, in a few cases of common catarrh, in diarrhœa, and also (as I shall have occasion to shew in a future part of this Essay) in Indian Cholera.

"Acute bronchitis frequently affords an excellent example of simple inflammation of a mucous membrane, without increased secretion. In some cases of it, the secretions during the first stage are diminished; and then, an increased secretion from the inflamed membrane always relieves the disease.† In the first stage of gastritis, and of enteritis, the secretions of the gastro-enteritic mucous membrane are generally diminished; and these diseases also are relieved by an increased flow of the natural secretions of the membranes. These examples sufficiently prove, that inflammation may occur alone in a mucous membrane, without being accompanied by increased secretion.

"There are also numerous examples of catarrh without inflammation. Common catarrh of the air passages, without inflammation, is familiar to every one. Diarrhœa generally does not exhibit the slightest symptom of inflammation; and in it, the secretion of the enteric mucous membrane is increased and vitiated." 11.

Our author next proceeds to consider the state of the general system, induced by these two local affections respectively. Acute inflammation is accompanied by increased force of the circulation, whilst, in catarrh, sufficiently severe to have any influence on the general sanguiferous system, the skin will be found cold, and the pulse at the wrist small. This diminution of size in the superficial arteries, and coldness of the surface, he is disposed to attribute, not to a diminution of the force of these arteries, but to that of the quantity of blood circulating through them, from its derivation, if we may so speak, to the interior, caused by the catarrhal affection of the mucous membranes. It is subsequently admitted, that inflammation and catarrh mutually excite, or pass into each other.

After quoting M. Billard, to show that the healthy colour of the gastro-enteritic mucous membrane in the adult, is white, or greyish white; and that in those who die during digestion, the mucous membrane of the stomach, duodenum, and upper part of the jejunum, is of a light rose colour, he details some experiments on dogs, of considerable interest, illustrative of the effect of certain medicines on this membrane. The inference apparently deduced from them is, that the sudden or transitory action of tartrate of antimony, corrosive sublimate, and calomel, induces catarrh; whilst inflammation results from the longer continued application of the same medicines. One experiment only is

* "This is the opinion of LAENNEC. Vide De L'Auscultation Mediate, T. 2. p. 65. It also appears to be BROUSSAIS' opinion."

† "Vide HASTINGS' Treatise on Inflammation of the Mucous Membrane of the Lungs, p. 161."

narrated, in which opium was administered; and so far as any inference can be drawn from a single observation, its tendency is to induce inflammation and not catarrh.

We are furnished with the following concise summary of this first division of the work.

"1. Mucous membranes are liable to two distinct simple morbid affections, viz. inflammation and catarrh.

"2. Catarrh consists of a diseased action of the secretory apparatus of a mucous membrane, which produces an increased and vitiated secretion; and is characterised by the membrane in which it occurs being generally whiter than natural, and by the quantity of the blood towards the surface of the body being diminished.

"3. Either of these morbid affections may occur alone in a mucous membrane, or conjoined with the other.

"4. Some medicines produce an inflammatory, others a catarrhal action, in mucous membranes: and a long continued action of certain medicines produces the former, while a short continued action of the same medicines produces the latter effect.

"5. There is no direct sympathy between the skin and liver; and the action of the liver and many other glands is much influenced by the condition of the mucous membrane upon which their excretory ducts open." 42.

The next, certainly the most interesting, section of the work, is on the autopsy of the disease. After pointing out the habitual neglect of the mucous tissues in our pathological investigations, and relating an anecdote illustrative of it, he presents us with the following results of his researches.

"The morbid appearances that are *invariably* met with in Cholera are confined to the mucous system; those observed in other systems being only occasional. In all the dissections I have made, the following appearances have been present. A whitish, opaque, viscid substance was found adhering to the surface of some portions of the mucous membranes; and in many cases it was so abundant in the intestines as completely to fill parts of them of a greater or smaller extent. The stomach and portions of the intestines were filled with a transparent or turbid serous fluid; and, frequently, the viscid matter mentioned above was found intimately mixed with the serous fluid, or floating in it in the form of flakes. The mucous membranes (except when inflamed) had an unnatural whiteness; were frequently soft and pulpy; and in general (especially in the stomach and small intestines) could be easily detached by scraping, in the form of a thick pulp, from the subjacent coat. These appearances were sometimes more or less partial; but some of them were generally found throughout the whole extent of the alimentary canal. They extended, in some cases, to the mucous membrane of the bladder and ureters; and were found, in two or three instances, in the pulmonary mucous membrane." 47.

"The morbid appearances that have been found next in frequency to those already mentioned, are, venous congestion in the viscera, particularly in those of the abdomen; dark-coloured blood in the veins, and sometimes in the left side of the heart; and inflammation in some part of the mucous membranes. I have generally found inflammation (when present at all) confined to the pyloric extremity of the stomach and small intestines. I have also met with many cases in which no inflammation could be detected." 48.

The blood drawn has been perfectly black, of the consistence of liquid honey, or forming a uniform coagulum after a few minutes exposure to the air; and these appearances it has retained for twenty four hours without separating into serum and crassamentum. In some cases, though darker than usual, it has furnished a good deal of serum on coagulating. Occasionally, there has been no morbid appearance of the circulating fluid.

The cholera secretion consists of two substances, the one a transparent serous fluid, the other an opaque white coagulum; the former perfectly soluble in cold water, the latter quite insoluble. These matters were submitted to the action of re-agents with the following results:

"1. SEROUS FLUID.

"*a.* Tincture of galls produced a precipitate, when added to a mixture of the serous fluid with cold water.
"*b.* Alcohol produced a precipitate when added to the same mixture.
"*c.* Muriate of mercury produced a white precipitate.
"*d.* Sulphuric acid produced a white precipitate.
"*e.* It was coagulated by heat.
"*f.* It did not affect litmus paper.

"2. COAGULATED MATTER.

"*a.* Insoluble in cold water.
"*b.* Slightly soluble in boiling water.
"*c.* Dissolved when boiled in acetic acid.
"*d.* Dissolved by pure aqua ammoniæ.
"*e.* Not changed when triturated with calomel.
"*f.* Prussiate of potassa, when added to the solution *c*, produced a copious yellow precipitate.

"The first set of these experiments proves, that the fluid part of the secretion is pure serum, which is particularly confirmed by *d* and *e*. The second set proves, that the coagulated part of the secretion is fibrin; test *f* being that which, according to Berzelius, particularly distinguishes that substance. The secretion, therefore, has a composition similar to that of blood, deprived of its colouring matter; but the proportions of the serum and fibrin in the secretion are, I imagine, seldom the same as those we find in blood; for, in most cases of Cholera, there is an enormous quantity of the serum thrown out by the stomach and intestines, with only a small quantity of coagulated matter." 53.

These conditions of the tissue itself, and its secretion, are not confined, in severe cases, to the gastro-enteritic, but extend to the bronchial and genito-urinary mucous membrane.

The whole of this section of the work is valuable, and highly creditable to the author; but we do not consider that which follows, in which he is occupied, not in observing, but in explaining phenomena, equally felicitous. The shrunk features, cold skin, and small pulse, he ascribes very plausibly to the afflux of blood to the interior; but when he descends to certain subordinate details, we find him apparently at fault. Profuse perspiration, we are told, sometimes exists when the pulse at the wrist is small, or altogether imperceptible; and this, he insists, is not to be ascribed to debility nor to sympathy between the mucous tissue and the skin; but to the simultaneous existence of an analogous morbid affection of that membrane and the surface of the body. Now it seems but reasonable to expect, that if a disease similar to that which produces such an afflux to the interior existed simultaneously on the surface, there would be a division of the flood between the centre and the periphery, and instead of smallness or imperceptibility of the radial pulse, we should expect to find it tolerably forcible. The dark colour of the blood in this disease he is disposed to ascribe very properly to its not being decarbonized in its passage through the lungs, and quotes from Dr. Davy the interesting facts, that the air expired by cholera patients is colder, and contains less carbonic acid than usual, in proof of this explanation. But he seems scarcely warranted in ascribing this defective decarbonization to the presence of diseased secretion in the bronchia, since he admits that the blood is frequently dark when there is no accumulation of mucus there. Were we to venture on suggestion, we should refer to the influence which Mr. Brodie's experiments have proved that the nervous system possesses over calorification, and the changes which the blood undergoes in the pulmonary and capillary vessels, as being most likely to furnish its real explanation. But Dr. Christie is disposed to regard the nervous system as a sort of King Log, who must not be allowed any participation in state affairs—nay, so great is his contempt for it, that, backed by a reviewer in our worthy contemporary, the Edinburgh Journal, he has bestowed on Mr. Orton, who wrote on cholera, a sound rating for having attempted to invest this *quondam* autocrat of all the functions with a little actual authority. The author subsequently attempts to found his explanation on the disordered state of the delicate membrane lining the air-cells; but this is purely hypothetical, since this disordered state can be inferred only from the presence of the mucous secretion. The other physical peculiarities of the blood, such as its remaining perfectly fluid, or, if it do coagulate, its throwing out no serum, are explained in a manner so perfectly speculative, that to relate it could afford no interest to our readers. We are rather surprised that an individual, so accurate in his researches as Dr. Christie, made no experiments of an analytical nature on these morbid fluids.

The practice recommended by our author seems much the same as that generally adopted in this too often fatal malady: bleeding, counter-irritation by blisters, sinapisms, or the boiling water blister, internal stimuli, calomel and opium. The apparent inconsistency of exhibiting calomel, a medicine which induces intestinal catarrh, in a disease of which such catarrh is the very essence, is attempted to be obviated by investing it with the additional power of correcting, while it increases, secretion. The whole of this portion of the work strongly reminds us of what we frequently observe in medical writings: new pathological views are advanced, supported by hosts of facts, and demonstrated by overwhelming arguments; but when we come to the therapeutics, instead of more appropriate remedial measures founded on these novel doctrines, we find an adaptation of the doctrines to the existing practice. This certainly tends to show that sound therapeutics occasionally outrun what are deemed correct pathological views; which may, perhaps, lead to the supposition, that our practice is more frequently directed by tolerably obvious phenomena than recondite causes. These remarks are not made with the intention of discouraging morbid anatomy, for the zealous culture of which we are warm advocates; neither does it appear to us that they furnish any serious objection to it. In every branch of science accurate ideas are of value, because they gratify a laudable feeling of the human mind: precise knowledge is an abstract good. But besides, we do not at once perceive the ultimate bearings of a new fact: that which is deemed fruitless to-day, may to-morrow be found to admit a useful application. To illustrate this by the case before us, should Dr. Christie's views of the distinction between inflammation and catarrh be confirmed by the observations and reasonings of others, it may throw considerable light on the nature and treatment of various diseases, though the author, having found the practice in cholera what he thought good, has left it undisturbed by his peculiar opinions. But we would suggest to pathologists, that the sooner they apply their discoveries the better; for we are sufficiently acquainted with the *useful* character of the British professional public to be convinced that they will not long concern themselves about *tints* of membranes which are to impart no *hue* to their practice.

We have intimated, and we hope Dr. Christie will excuse us when we now positively assert, that the opinion of the non-inflammatory nature of catarrh, on which alone he can found any claim to originality, requires confirmation by the researches of others. The fact on which the opinion mainly rests, the white colour of the mucous membrane, would not be esteemed conclusive, for many writers have held, that whiteness after death is no evidence that redness did not exist during life; and with some pathologists of the highest character, both at home and abroad, the softened pulpy state of the membrane would be considered a proof that it had undergone the process of inflammation. Though we have thus expressed our doubts of the security of the very key-stone of his work, yet we would, by no means, have it supposed that we think lightly of Dr. Christie's merits. We consider his book indicative of patience of research, experimental ingenuity, and general sobriety of induction; and we recommend its attentive perusal to our professional brethren, especially to those who may have occasion to visit the warmer latitudes. To say that some of the opinions expressed in it require further investigation is no more than may be predicated of all medical doctrines which possess any claim to novelty.

Dr. Christie writes with great perspicuity, the only quality of style worth regarding in medical publications. In one point only have we found any obscurity, and that is his nomenclature of colours. We learn, for instance, that the gastro-enteritic mucous membrane of the dog has a light rose or white colour; and the mucous membrane of the stomach and large intestines is said to be of a deeper *rose* colour than that of other parts of the alimentary canal. But in the experiments on dogs, *rose* colour of the stomach is considered a proof of inflammation. He quotes, apparently in confirmation of his own views, M. Billard, as stating that the *healthy* colour of the gastro-enteritic lining is in the adult *white* or greyish white; but *white* is constantly adduced by him as evidence of his other pathological state, catarrh. There is an extreme difficulty in conveying accurately these simple ideas; and probably the best mode of surmounting it is that adopted by Laennec respecting the stethoscopic sounds, viz. to compare the sound, colour, &c. intended to be described, to that of some familiar object, if a suitable one can be found.

---

